# An Odorless, Tasteless, Absolute Anti-Septic; Salicylic Acid

**Published:** 1875-03

**Authors:** 


An Odorless, Tasteless, Absolute Anti-Septic; Salicylic Acid. The *Boston Medical and Surgical Journal*, Jan. 28, 1875, prints a letter from Professor Horsford, announcing, on the authority of Professor Schwartz, of Gratz, the production in large quantities of salicylic acid, the most perfect disinfectant known. It is without odor, tasteless, not poisonous, and, even in small quantities, absolutely preventing putrefaction. Meat immersed in a solution of salicylic acid, in an open vessel, remained perfectly sweet for weeks. It prevents milk from coagulation. Fruits do not become mouldy, and wounds heal without festering. In the case of a patient whose leg was amputated, the wound was sprinkled with a little powdered salicylic acid, and bandaged for six days without being touched; it was then found to be healed over without the slightest formation of pus. It can be imported in small quantities through the mail and in large quantities through any of the leading druggists. Its present price is not far from three dollars per ounce.

Removal of Foreign Bodies from the Ear.—Dr. John Cleland suggests that, in removing foreign bodies from the ear, the point of the probe or needle used for extraction should be placed *below* the object to be dislodged. By so doing it is placed between two inclined planes, and is readily and easily expelled.—*Phila. Med. Times*, Jan. 23, 1875, fr. *The Lancet*, Dec. 5, 1874.

